# Development and Preliminary Evaluation of the Effects of an mHealth Web-Based Platform (HappyAir) on Adherence to a Maintenance Program After Pulmonary Rehabilitation in Patients With Chronic Obstructive Pulmonary Disease: Randomized Controlled Trial

**DOI:** 10.2196/18465

**Published:** 2020-07-31

**Authors:** Begoña Jiménez-Reguera, Eva Maroto López, Shane Fitch, Lourdes Juarros, Marta Sánchez Cortés, Juan Luis Rodríguez Hermosa, Myriam Calle Rubio, María Teresa Hernández Criado, Marta López, Santiago Angulo-Díaz-Parreño, Aitor Martín-Pintado-Zugasti, Jordi Vilaró

**Affiliations:** 1 Departamento de Fisioterapia Facultad de Medicina Universidad San Pablo-CEU, CEU Universities Madrid Spain; 2 Lovexair Foundation Madrid Spain; 3 Hospital Universitario 12 de octubre Madrid Spain; 4 Hospital Clínico San Carlos Madrid Spain; 5 Hospital Universitario de La Princesa Madrid Spain; 6 Ramon Llull University Barcelona Spain

**Keywords:** adherence, pulmonary rehabilitation, mHealth, COPD, chronic obstructive pulmonary disease

## Abstract

**Background:**

Pulmonary rehabilitation is one of the main interventions to reduce the use of health resources, and it promotes a reduction in chronic obstructive pulmonary disease (COPD) costs. mHealth systems in COPD aim to improve adherence to maintenance programs after pulmonary rehabilitation by promoting the change in attitude and behavior necessary for patient involvement in the management of the disease.

**Objective:**

This study aimed to assess the effects of an integrated care plan based on an mHealth web-based platform (HappyAir) on adherence to a 1-year maintenance program applied after pulmonary rehabilitation in COPD patients.

**Methods:**

COPD patients from three hospitals were randomized to a control group or an intervention group (HappyAir group). Patients from both groups received an 8-week program of pulmonary rehabilitation and educational sessions about their illness. After completion of the process, only the HappyAir group completed an integrated care plan for 10 months, supervised by an mHealth system and therapeutic educator. The control group only underwent the scheduled check-ups. Adherence to the program was rated using a respiratory physiotherapy adherence self-report (CAP FISIO) questionnaire. Other variables analyzed were adherence to physical activity (Morisky-Green Test), quality of life (Chronic Obstructive Pulmonary Disease Assessment Test, St. George’s Respiratory Questionnaire, and EuroQOL-5D), exercise capacity (6-Minute Walk Test), and lung function.

**Results:**

In total, 44 patients were recruited and randomized in the control group (n=24) and HappyAir group (n=20). Eight patients dropped out for various reasons. The CAP FISIO questionnaire results showed an improvement in adherence during follow-up period for the HappyAir group, which was statistically different compared with the control group at 12 months (56.1 [SD 4.0] vs 44.0 [SD 13.6]; *P*=.004) after pulmonary rehabilitation.

**Conclusions:**

mHealth systems designed for COPD patients improve adherence to maintenance programs as long as they are accompanied by disease awareness and patient involvement in management.

**Trial Registration:**

ClinicalTrials.gov NCT04479930; https://clinicaltrials.gov/ct2/show/NCT04479930

## Introduction

### Pulmonary Rehabilitation in Chronic Obstructive Pulmonary Disease

Chronic obstructive pulmonary disease (COPD) is a chronic, preventable, and treatable disease process characterized by airflow limitation that is not fully reversible [1]. It is a major public health problem because it represents a high health cost due to the direct and indirect expenses it generates, including the significant consumption of resources and medical and pharmaceutical services, as well as the demand for support and social assistance arising from sickness from work [2-4].

The benefits of pulmonary rehabilitation are such that it has been compared favorably with other strategies, such as drug treatment or telemedicine, in terms of its cost-effectiveness. Thus, it is well known that it can reduce the use of health resources and promotes a reduction in COPD costs [5-7].

Pulmonary rehabilitation is defined as “a comprehensive intervention based on a thorough patient assessment followed by patient tailored therapies that include, but are not limited to, exercise training, education, and behavior change designed to improve the physical and psychological condition of people with chronic respiratory disease and to promote the long-term adherence to health-enhancing behaviors” [8].

However, despite evidence of the benefits provided by pulmonary rehabilitation, these do not last over time, disappearing progressively between 6 and 12 months after the end of rehabilitation, with patients having values even lower than those presented pre–pulmonary rehabilitation [9]. The lack of adherence to maintenance programs seems to be one of the possible causes explaining the loss of health benefits [10,11], so the creation of effective strategies to increase adherence to such programs is the key to maintaining the effects achieved after pulmonary rehabilitation. Aspects such as self-management, patient empowerment, and the acquisition of co-responsibility in therapy are the focus of current research, as they seem to guide determinant behavioral changes to maintain disease control.

### Telehealthcare as a Solution

Mobile health (mHealth) systems in COPD, designed according to the needs of the patients, aim to improve adherence to maintenance programs by promoting the change in attitude and behavior necessary for patient involvement in the management of the disease [12,13].

Both patients and professionals recognize the importance of designing individualized mHealth interventions that encompass the different aspects associated with the disease and facilitate self-control through appropriate feedback for each dimension, without replacing or dominating the patient’s decision. Health informatics platforms should not replace, at any time, the personal and regular relationship with health care professionals but complement it, as it is very important to maintain continuous and open contact with the multidisciplinary team that provides the necessary support and attention. But, in any case, patients should be responsible for their care, which may lead to an improvement of the therapy’s effectiveness [12].

The follow-up programs designed and analyzed so far are not effective, as they do not achieve patient adherence to them, showing a high dropout rate. There is also great controversy regarding the methods used, types of mHealth systems, specific design of maintenance programs, and duration and frequency of follow-up. Most of the studies consulted highlight the influence of the biopsychosocial context of the individual on the involvement with their illness and treatment [14-16].

As stated above, and due to the improvement in adherence to the treatments that digital platforms promote, this study aimed to evaluate whether an mHealth web-based platform (HappyAir) would improve adherence to a 1-year maintenance program applied after pulmonary rehabilitation in COPD patients.

## Methods

### Study Design and Clinical Trial Protocol

The initial objective of the study was the development of a clinical tool, the HappyAir system, which included the web-based platform and mobile app to allow its use by COPD patients for the management of their long-term pathology. As previous studies have found a possible reluctance in many COPD patients toward new technologies [17], it was decided to generate a new web-based app with an intuitive design and easy operation in order to avoid this possible reticence. The HappyAir system was then integrated into the long-term follow-up program of the intervention group. The completion of the protocol at different stages of the preliminary evaluation study, some not initially planned, meant that the clinical trial was not registered in due course.

A multicenter, longitudinal, prospective, randomized controlled clinical trial was conducted on 44 COPD patients who underwent an integrated care plan monitored with mHealth. The recruitment and follow-up were carried out between December 2015 and May 2017. Patients, who were recruited from participating hospitals, underwent a randomization process, establishing two groups: the intervention group (HappyAir) and control group (see later randomization procedure section).

The study was conducted in two stages, with a total duration of 12 months. The first stage corresponded to the 8-week pulmonary rehabilitation program that was conducted on both groups at the hospital. It included several procedures that followed the guidelines of the Spanish Society of Pulmonology and Thoracic Surgery and are included in hospital protocols, such as muscle training, respiratory physiotherapy, and education on relevant aspects of chronic respiratory disease [18].

The second stage corresponded to a 10-month follow-up period. Patients from both groups underwent a maintenance follow-up community-based program at home and in the neighborhood, in which they were advised to perform physical activity and breathing exercises daily. Patients assigned to the HappyAir group followed an integrated care plan using a mobile device with the pulmonary care web-based app (HappyAir app) and were instructed in its use (Multimedia Appendix 1). The control group only went to the hospital for the scheduled evaluations during the follow-up period, without receiving integrated supervision or using the HappyAir app.

**Figure 1:**
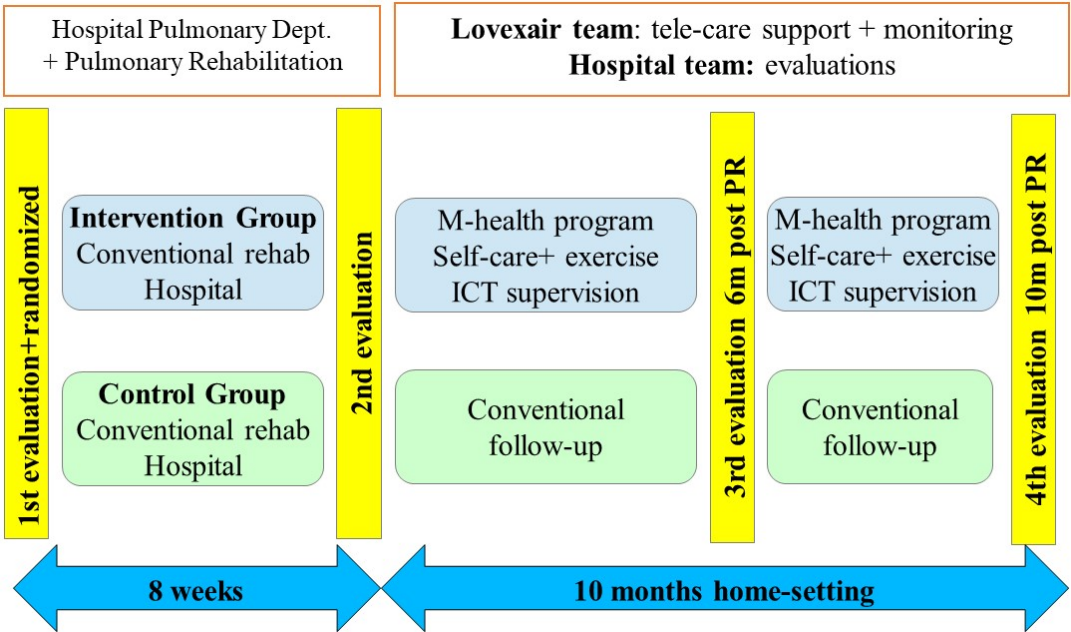
Intervention and control group process and follow-up.

In order to ensure the correct follow-up of the two populations studied, four evaluations were completed by a blinded assessor: at baseline (pre–pulmonary rehabilitation), immediately after pulmonary rehabilitation (post–pulmonary rehabilitation), after 6 months of follow-up, and after 12 months from the beginning of the study (10-month postrehabilitation follow-up; Figure 1).

The study protocol was approved by the ethics committees of 12 de Octubre University Hospital (No.15/308), La Princesa University Hospital, and San Carlos Clinical University Hospital (16/111-E). All patients gave written informed consent to participate in the study.

### Study Population and Recruitment

Patients were recruited by convenience sampling through face-to-face interviews at participating hospitals. The recruitment of subjects was performed from patients attending pneumology consultations at the rehabilitation service of the hospitals participating in the study. The participants belonged to the geographical area of Madrid.

COPD patients were selected according to the following inclusion criteria: COPD patient, aged between 55 and 85 years, with degree of severity II, III, or IV of the Global Initiative for Chronic Obstructive Lung Disease (GOLD) scale, in a stable clinical situation (no exacerbations in the last 6 weeks).

Exclusion criteria were patient with unstable cardiovascular disease or muscular, osteoarticular, auditory, visual, or central or peripheral nervous system impairment that prevented the performance of the rehabilitation program or evaluation tests or cognitive impairment that made it difficult to understand the education program and manage the HappyAir system.

After participants were informed about the aim and characteristics of the investigation, they were asked to carefully read and sign an informed consent to be able to participate in the study.

### Pulmonary Care Web-Based App (HappyAir App)

The HappyAir app comprises two main parts: an educational program providing patients useful information and advice about their illness and data collection related to physical activity and disease. The HappyAir app reminded the HappyAir group daily to use the app, indicating that they record medication intake, daily exercise time (minutes), level of tiredness after the exercises (good, little tired, very tired, or exhausted), and daily mood (happy, little sad, sad, or very sad). These records allowed for usability evaluation of the HappyAir app. During development, a focus group of patients, health care professionals, and app developers met to detect usability problems and adapt the app to the characteristics of the target group.

After patients finished pulmonary rehabilitation, we offered an education session that consisted of 3 to 4 hours of practical class demonstration plus an online support aid; the handling of the device and the web-based app were explained so the patients could become familiar with them.

The therapeutic educators had access to the platform to supervise the evolution of the HappyAir group during the follow-up period, collect data, see the results of clinical evaluations, record weekly and monthly goals, and contact the physician responsible for the patients (in case they detected warning signs of possible exacerbations or relapses). In addition, the pulmonologist and physiotherapist of the hospital had access, by login, to the data collection platform to enter the clinical data, record results of the evaluations, see the evolution of patients, and contact the therapeutic educator.

The HappyAir integrated plan was designed as a model of a therapeutic program based on communication that introduced the figure of the therapeutic educator (physiotherapist or respiratory coach) in order to design interventions focused on the patients and their needs, with minimal intervention and presence, making the patients responsible for their self-care and management of their illness. Patient and educator shared responsibility.

### Outcome Measures

#### Treatment Adherence

Adherence to the maintenance program was measured with the respiratory physiotherapy adherence self-report questionnaire (CAP FISIO) questionnaire administered by a blinded assessor at each follow-up session in the hospital. This is a questionnaire created to assess the adherence to physiotherapy treatments [19]. We adapted some terms of the questionnaire to the purpose of this study, because to our knowledge, no questionnaire existed that measured adherence and perception in pulmonary rehabilitation for chronic respiratory disease. It consists of a total of 16 items, with a Likert scale to score each one, with 1 point as totally disagree and 4 points as totally agree. Three different dimensions of results are obtained: total score, perception, and adherence. The final rating scale is set to a range of minimum of 16 points to a maximum of 64 points. A higher final score reflects better adherence to the intervention. The internal consistency was set with a Cronbach alpha [19].

Adherence to physical activity was measured with the therapeutic compliance questionnaire (Morisky-Green Test), administered by a blinded assessor at each follow-up session in the hospitals. This method, which has been validated for various chronic diseases, was originally developed by Morisky et al [20] to assess medication compliance in patients with high blood pressure [21]. Since the test was introduced, it has been used in the evaluation of different diseases. It consists of a series of 4 contrast questions with a yes/no dichotomous answer, reflecting the patient’s behavior with respect to compliance. In order to consider the patients compliant with or adherent to treatment, the first and last two answers must be no and the second answer must be yes. Answering at least one of the questions incorrectly indicates poor adherence. Given the scarcity of published questionnaires for the evaluation of adherence to physical exercise of patients with chronic respiratory disease, it has been decided to adapt an already validated test (as described earlier, Morisky-Green Test) to verify adherence in our study. To do this, the word medication was change to physical exercise.

#### Quality of Life

Quality of life was measured with three different self-reported questionnaires administered by a blinded assessor at each follow-up session in the hospitals.

##### Chronic Obstructive Pulmonary Disease Assessment Test

The Chronic Obstructive Pulmonary Disease Assessment Test (CAT) is a self-administered questionnaire that measures the impact of COPD on the patient’s quality of life. It consists of 8 items, each of which has 5 possible answers from 1 (being absence of symptoms) to 5 (being the worst possible situation). The final score scale is set in a range of 8 to 40 points. A difference of 2 points or more would represent a clinically significant difference in pre- and posttreatment health status. The difference between stable status and exacerbation is a 5-point increase on the 40-point scale. Higher scores mean greater deterioration in COPD-related quality of life [22].

##### St. George’s Respiratory Questionnaire

The St. George’s Respiratory Questionnaire (SGRQ) is a validated questionnaire that measures quality of life related to health or perceived health in COPD patients. It consists of 50 items divided into 3 dimensions: symptoms (frequency and severity of symptoms; 8 items), activity (limitation of activity due to dyspnea; 16 items), and impact on daily life (psychological and social functioning disorders; 26 items).

The final score scale is set in a range from 0 (no limitation of the quality of life) to 100 (maximum limitation of the quality of life). A difference of 4 points is considered a clinically significant difference [23,24].

##### EuroQOL-5D

The EuroQOL-5D is a questionnaire that measures quality of life related to health or perceived health, which, unlike the SGRQ, is simpler to administer. Therefore, it was also decided to use it in case there was not much acceptance of the SGRQ, which, despite being very complete and extended in its application, is a very long questionnaire.

The EuroQOL-5D consists of 5 items divided into 5 dimensions: mobility, self-care, habitual activities, pain or discomfort, and anxiety or depression. It has a minimum number of levels (3) for each dimension (1=no problem, 2=some problem or moderate, and 3=many problems). Preferably, the questionnaire should be self-administered, although administration by personal interview or by mail has been shown to be acceptable. It generates an index that allows the evaluation of health conditions. The state of health of the individual is defined as the combination of the level of problems described in each of the 5 dimensions, using a 5-digit number that reflects the value of each dimension [25,26].

##### Exercise Capacity

Exercise capacity was measured with the 6-Minute Walk Test (6MWT). This is a simple test in which the subject must walk in a circuit straight and without irregularities, at least 30 meters, for a period of 6 minutes, with the aim to reach the maximum possible distance (walking as quickly as possible, without running). This test was performed following the protocol established by the American Thoracic Society in 2002, according to the 2014 update [27].

### Randomization Procedure

We used a computer-generated simple randomization procedure, using the online randomization tool Research Randomizer (Geoffrey C Urbaniak and Scott Plous). Before the beginning of the study, distribution was made in two groups through the Research Randomizer program, and a list of patients designated to each group was drawn up, considering a homogeneous distribution of groups for each hospital. This listing was sequentially numbered and coded to ensure the confidentiality of participants and masking of the professionals who performed the rehabilitation protocol.

Patient data collected during the study were documented anonymously and dissociated and linked to an ID code (patient identification number) so that only the hospital investigator could associate such data to an identified person. The principal researcher of the study, external to the hospital, established the relationship between the ID provided by hospital staff with the code assigned in the tracking platform.

The follow-up assessment of outcome measures of both groups was carried out by a blinded assessor. Due to the characteristics of the intervention, health care professionals and patients could not be blinded to the group assignment.

### Sample Size

For the calculation of the sample size to test the difference between the treated and control groups in the total score variable after 12 months of follow-up (the main objective of the study), a pilot study was conducted with 7 individuals in each group, with the following results: in the control group, the mean was 44.2 (SD 12.8), while in the HappyAir group, the mean was 55.6 (SD 6.2). With these results and using the G*Power version 3.1.9.4 program (Heinrich-Heine-Universität Düsseldorf) with a 2-sided test of an alpha level of .05 and a power of 80%, it was determined that 14 individuals would be needed in each group. Assuming the probability of dropouts of 20% because of the long-term intervention, we decided to recruit the maximum number of patients available.

### Statistical Analysis

Statistical analyses were performed using SPSS Statistics 25.0 statistical software for Windows (IBM Corporation). Thus, a descriptive study of absolute and relative frequencies and distributions for each of the qualitative variables was completed. The normal distribution of the quantitative variables was demonstrated using the Kolmogorov-Smirnov normality test when the number of data exceeded 50 and the Shapiro-Wilk test when the number of data were fewer than 50.

Subsequently, the existence of statistically significant differences over time or in the different measurements of the quantitative variables was analyzed, and for this purpose, the *t* test was used for repeated measurements and the Wilcoxon test in the nonparametric or qualitative case.

The statistical significance of the intergroup and intragroup comparisons at all levels of segmentation was analyzed using parametric tests (*t* tests [for 2 samples], analyses of variance, and Welch tests) and nonparametric tests (Mann-Whitney *U* and Kruskal-Wallis tests), according to the distribution of the sample.

## Results

### Patient Characteristics

**Figure 2:**
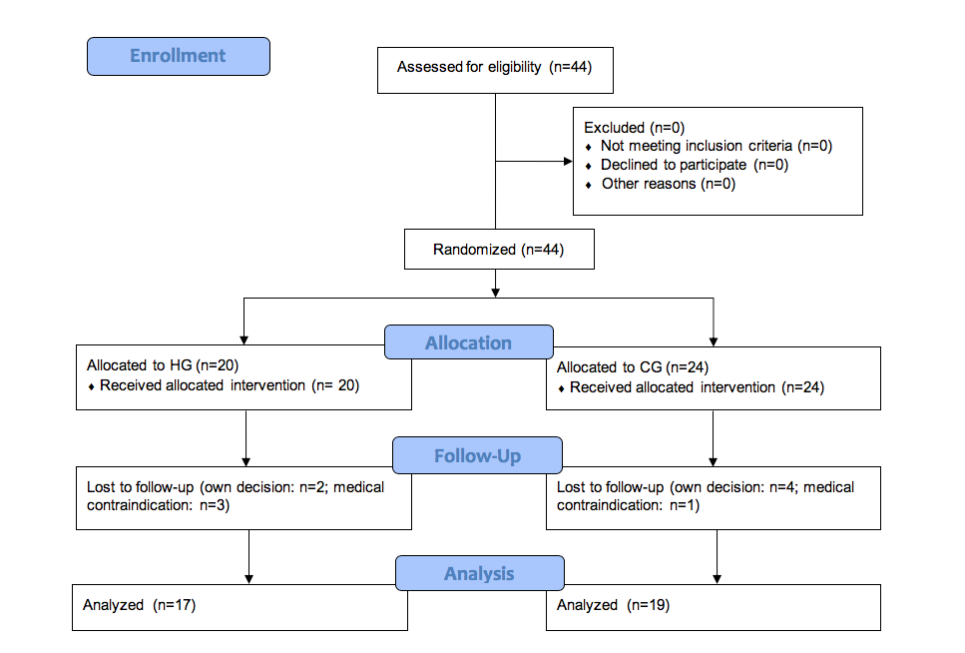
Distribution of patients Consolidated Standards of Reporting Trials flow diagram.

A total of 44 patients diagnosed with COPD were included in the study, randomly assigned to the control group or HappyAir group; among them, 8 patients dropped out of the study for different reasons. In the end, 36 patients completed the 12-month follow-up process and were included in the final analysis (22 men and 14 women; median age 68.11 [SD 6.74] years). A flowchart of participants in the study is shown in Figure 2. Demographic and baseline characteristics are shown in Table 1. None of the variables showed differences between the groups at baseline.

**Table 1:** Baseline and demographic characteristics of the study population.

Characteristic		Control group	Intervention group	*P* value
Age in years, mean (SD)		68.1 (7.0)	68.1 (6.6)	.97
**Gender**				.34
	Male, n (%)	13 (59.1)	9 (40.9)	—^a^
	Female, n (%)	6 (42.9)	8 (57.1)	—
Weight (kg), mean (SD)		68.4 (15.3)	70.1 (10.1)	.68
Height (cm), mean (SD)		162.9 (9.8)	161.7 (6.7)	.91
BMI (kg/m^2^), mean (SD)		26.07 (4.2)	26.50 (4.1)	.77
**GOLD^b^ classification**				.28
	Level II	2 (1M/1F)	3 (1M/2F)	—
	Level III	14 (10M/4F)	9 (4M/5F)	—
	Level IV	3 (2M/1F)	5 (4M/1F)	—
FEV_1_^c^ (%), mean (SD)		43.1 (13.6)	45.0 (15.3)	.95
FVC^d^ (%), mean (SD)		72.6 (24.4)	78.6 (22.9)	.44
FEV_1_/FVC (%), mean (SD)		44.5 (12.2)	49.06 (12.0)	.81
Oxygen users, n (%)		10 (52.6)	9 (52.9)	.52
Oxygen hours per day, mean (SD)		10.8 (10.8)	10.0 (11.2)	.98
Smokers, n (%)		5 (26.3)	3 (17.6)	.43
Exsmokers, n (%)		11 (57.9)	11 (64.7)	.77

^a^: not applicable.

^b^GOLD: Global Initiative for Chronic Obstructive Lung Disease.

^c^FEV_1_: forced expiratory volume in the first second of expiration.

^d^FVC: forced vital capacity.

### Adherence

The results of adherence to the maintenance program measured with the CAP questionnaire and adherence to physical activity using the Morisky-Green questionnaire are detailed in Table 2.

#### Cuestionario Adherencia Percepción Questionnaire

**Figure 3:**
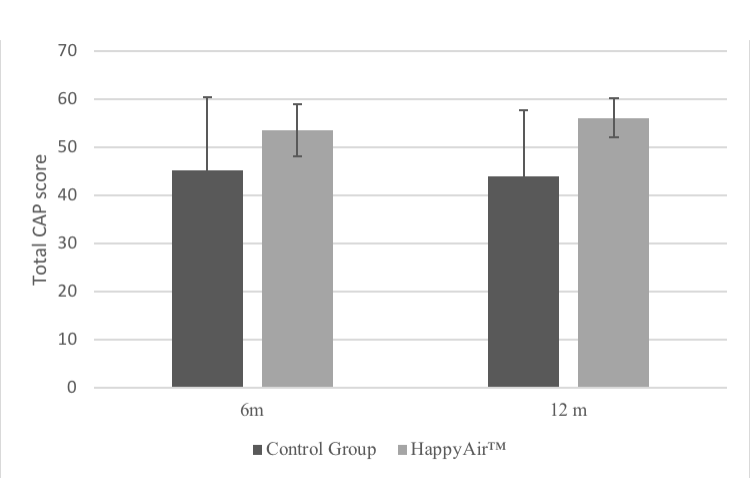
Adherence to HappyAir program: total dimension of CAP questionnaire.

Total CAP dimension results showed an improvement in adherence during the follow-up period, which was statistically significant at 12 months after pulmonary rehabilitation. Differences observed at 6 months were 45.3 (SD 15.0) and 53.6 (SD 5.4) in the control group and HappyAir group, respectively. Differences at 12 months were 44 (SD 13.6) and 56.1 (SD 4.0) in the control group and HappyAir group, respectively. The results showed significant differences between the groups at 12 months (*P*=.004; Figure 3).

#### Morisky-Green Questionnaire

**Figure 4:**
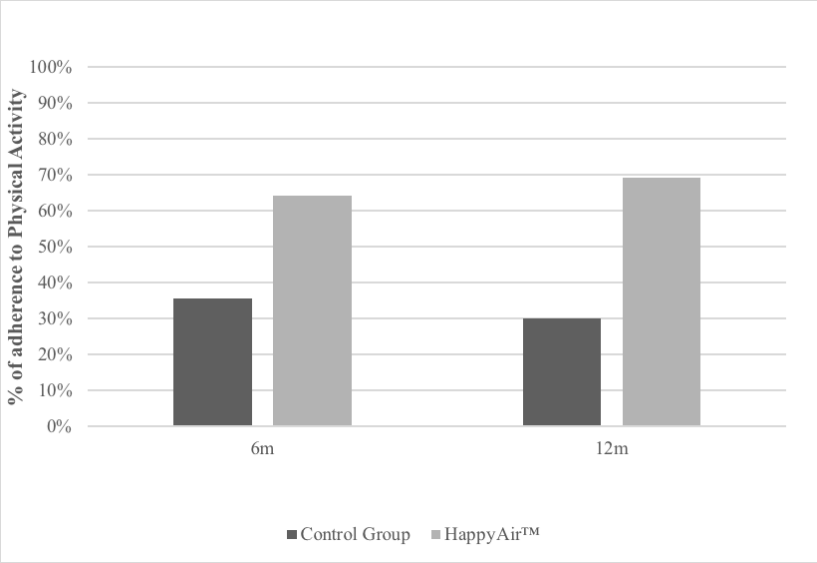
Percentage of patients adherent to physical activity: Morisky Green questionnaire.

In the HappyAir group, 25% of patients were adherent at 12 months of follow-up versus 11% of patients in the control group. The intergroup factor analysis showed statistically significant differences in adherence to physical activity at 12-month follow-up (*P*=.049). The intragroup factor analysis of exercise adherence showed no statistically significant differences over time (Figure 4).

**Table 2:** Adherence and perception effects of the home rehabilitation program.

Variable		Control group (n=19), mean (SD)		HappyAir group (n=17), mean (SD)			Intragroup *P* value (95% CI), 12 m^a^ vs 6 m		Intergroup *P* value		
		6 m	12 m		6 m	12 m	Control	HappyAir		6 m	12 m
**CAP^b^**											
	Total	45.3 (15)	44 (13.6)		53.6 (5.4)	56.1 (4)	.69 (–5.3 to 7.9)	.05 (–5.01 to 0.075)		.16	.004
	Adherence	21.7 (6.2)	21.1 (5.5)		24.7 (2.9)	25.7 (1.8)	.64 (–2.2 to 3.4)	.19 (–2.5 to 0.57)		—^c^	—
	Perception	23.5 (9)	22.9 (8.1)		28.9 (4.2)	30.4 (2.5)	.74 (–3.3 to 4.5)	.14 (–3.4 to 0.54)		—	—
**Morisky Green**											
	Adherence PA^c^ (%)	35.70	30.80		64.30	69.20	—	—		.11	.049

^a^m: month.

^b^CAP: Cuestionario Adherencia Percepción

^c^Not applicable.

^d^PA: physical activity.

### Usability of the HappyAir App

Most of the patients were able to start using the app and almost all in the HappyAir group managed to be skilled and 100% autonomous 15 days after beginning to use the platform. We observed that the average number of physical exercise records was 242 records per patient during the 10 months of follow-up, which is almost a daily record. Records showed that approximately 92% of patients from the HappyAir group exercised daily, which reflects the high rate of use of the app.

### Physical Activity, Mood, and Fatigue

**Figure 5:**
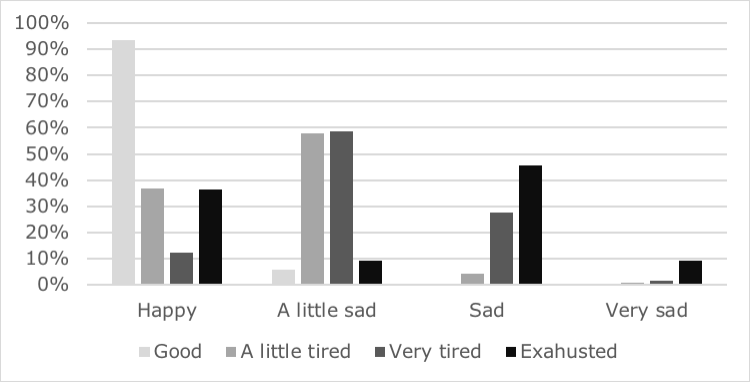
Relationship between mood state and physical activity.

Patients in the HappyAir group performed a mean of 66 (SD 37.43) minutes of daily physical activity (95% CI 65.03 to 67.21). Most of the patients analyzed showed a relationship between the feeling of tiredness experienced at the end of the exercises and mood, being less tired, in general, in those patients who were happy. Otherwise, when they finished tired, they felt sad or very sad. The Kruskal-Wallis test showed a significant effect (*P*=.001) between both variables (Figure 5).

### Quality of Life

Patients showed an improvement in quality of life in the CAT questionnaire, with a difference at 6 months during the follow-up period compared with a baseline of 2.2 (SD 0.3) and 4.2 (SD 0.4) in the control group and HappyAir group, respectively. This improvement was only significant in the HappyAir group (*P*=.001; 95% CI –6.6 to –1.6).

**Figure 6:**
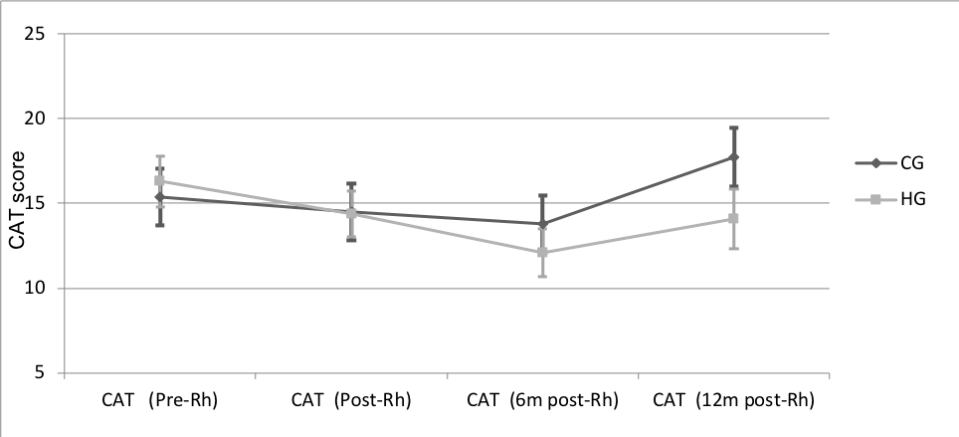
Quality of life: Chronic Obstructive Pulmonary Disease Assessment Test questionnaire.

No statistically significant differences between the two groups were observed in the CAT at 6 months (*P*=.53; 95% CI –5.8 to 3.1) or at 12 months (*P*=.21; 95% CI –8.1 to 1.9). However, the mean evolution graph (Figure 6) shows a difference of 3.6 points between the HappyAir group and control group after 12 months of follow-up, denoting a better quality of life in the HappyAir group with respect to the baseline evaluation, since a 2-point difference in the CAT questionnaire is established as the clinically relevant difference in quality of life outcomes.

Regarding the SGRQ, patients in the HappyAir group showed an improvement in quality of life after pulmonary rehabilitation and over time in symptoms, impact, and total dimensions but did not show significant differences in the activities dimension. No statistically significant differences between the two groups were observed in the SGRQ at 6 months (*P*=.77; 95% CI –10.2 to 13.9) or at 12 months (*P*=.79; 95% CI –13.2 to 17.0).

The differences in symptoms at 6 months follow-up compared to baseline were 15.8 (SD 3.3) and 12.1 (SD 2.2) in the control group and HappyAir group, respectively. These differences were significant both for the control group (*P*=.002; 95% CI –27.7 to 4.8) and HappyAir group (*P*=.04; 95% CI –23.8 to –0.2). The control group also showed differences in symptoms at 6-month follow-up after pulmonary rehabilitation compared with post–pulmonary rehabilitation results (14.9 [SD 2.1]; *P*=.01; 95% CI –26.9 to –1.6). The differences in impact at 12-month follow-up after pulmonary rehabilitation were 9.6 (SD 0.8) and 12 (SD 6.9) in the control group and HappyAir group, respectively, compared with baseline. These differences were only significant for the HappyAir group (*P*=.001; 95% CI –22.1 to –1.1). The control group also showed differences in impact at 12-month follow-up after pulmonary rehabilitation compared to results at 6-month follow-up (10 [SD 0.7]; *P*=.04; 95% CI –19.8 to –0.09).

Total dimension results showed an improvement in quality of life observed at both 6- and 12-month follow-up compared with baseline. The difference at 6-month follow-up was 3.2 (SD 1) and 6.4 (SD 1.2) in the control group and HappyAir group, respectively. Only differences in the HappyAir group were significant (*P*=.05; 95% CI –13.5 to 0.6). The difference at 12-month follow-up was 6.3 (SD 0.8) and 7.5 (SD 7.7) in the control group and HappyAir group, respectively. Only differences in the HappyAir group were significant (*P*=.04; 95% CI –16.1 to 1.2). The EuroQOL-5D questionnaire showed no statistically significant intragroup improvements and no statistically significant differences between groups (Multimedia Appendix 2).

### Exercise Capacity, Perceived Dyspnea, and Fatigue

Patients showed an improvement in walking distance after pulmonary rehabilitation, with differences in the 6MWT of 29 (SD 3) meters versus 42 (SD 14) meters in the control group and HappyAir group, respectively. Only differences in the HappyAir group were significant after pulmonary rehabilitation (*P*=.001; 95% CI 6.8 to 79.0). At 12-month follow-up, a decrease in walking distance was observed in both groups. However, the HappyAir group, although not statistically significant, was able to maintain a distance walked above the baseline values (Multimedia Appendix 2). No statistically significant differences between the two groups were observed at 6 months (*P*=.38; 95% CI –34.8 to 88.3) or 12 months (*P*=.58; 95% CI –47.6 to 82.6).

Regarding dyspnea after pulmonary rehabilitation, patients in both groups showed less dyspnea, with improvements of 0.4 (SD 0.5) and 1.1 (SD 0) in the control group and HappyAir group, respectively, which were not statistically significant (*P*=.05). Patients in both groups also showed less fatigue, with improvements at 6-month follow-up compared with a post–pulmonary rehabilitation of 0.2 (SD 0.7) versus 0.8 (SD 0.5) in the control group and HappyAir group, respectively, which were not statistically significant (*P*=.05). Lung function values showed no statistically significant differences between the two groups (Multimedia Appendix 2).

## Discussion

### Principal Findings

This study examined the effectiveness of a pulmonary care app designed to improve adherence to an integral 1-year maintenance program applied after pulmonary rehabilitation. The challenge was to design an effective intervention to maintain the effects obtained in the initial treatment. Our main hypothesis that the use of this web-based app would improve adherence to the maintenance program was confirmed, in addition to improvement in other variables such as quality of life, behavioral change, and adherence to physical activity.

### Adherence, Perception, and Mood

The key to making integral care plans in COPD work, either in a traditional way or through new technologies, is to consider in its design, in addition to the functional dimension, an emotional dimension [28]. This is because adherence is linked to a large number of affective factors such as personalized follow-up programs, social support, the patient’s state of mind, and even marital status [21,28-30], so it would be very interesting to take them into account to facilitate integration by patients.

Like other studies [31-33], our study showed a very positive perception of the integrated care plan in the HappyAir group compared with the control group, which is fully consistent with the adherence results in the same group. Those patients who believed that engaging in their treatment would help improve their health were active and responsible in managing their care plan. Their perception about the plan and its value in being continued over time was very positive, and they showed good physical ability, followed their daily care routines, had a positive attitude, and became confident in self-management, which was favorable to long-term adherence to their plan.

Thus, after analyzing the results of their adherence to the care plan using a questionnaire and the online web-based app that connected with their coach for additional support, we discovered both study groups, the control group and HappyAir group, progressed significantly at the beginning of the evaluation period with a positive trend in the engagement in their integrated care plan. What is significant in this study, is that, during the 10 months of monitoring, the control group no longer maintained that difference; however, in contrast, a clear improvement in adherence was observed in the HappyAir group.

These observations lead us to conclude that the integrated care plan, designed to improve adherence, managed to establish a more proactive and responsible attitude toward self-care and improved adherence. We believe that this holistic approach in integrated care with coach support is what has favored this important change in patients from a passive attitude to taking active responsibility in managing their care plan and following their treatment, a factor that is probably linked to behavior change and adherence. Therefore, remote care can be a very important and effective part of maintenance programs but only if it is accompanied by the behavioral change necessary to promote patient adherence [34].

Apart from that, it is important to consider COPD patients’ perceptions of the use of telehealthcare systems. Many patients encounter barriers, such as limited health literacy, difficulties in using technologies, and insufficient clinical support; they feel that mHealth systems could never completely replace face-to-face visits from a health care professional [35]. On the other hand, there are patients who consider technology a facilitator in the management of their disease, due to having a greater responsibility for their own health and for integrating personalized education [36,37].

In any case, when developing mHealth tools, it is important to consider factors such as age, information technology experience, education level, and possible comorbidities. Engagement with patients in the design and testing is essential to make sure the intervention is easy for older people [38-40].

### Relation Between Mood and Physical Activity Level

Mood has a strong influence on adherence, so enjoying therapy and overcoming depressive or anxiety states also facilitates success and patient involvement. The feeling of achievement improves self-efficacy [41,42]. Thus, in our study, the HappyAir group was monitored for 1-year using the web-based app. Daily exercise was recorded. This showed that the patients completed an average of 66.12 minutes of physical activity daily, even exceeding the recommendations of the World Health Organization and American College of Sports Medicine [43,44]: a minimum of 150 minutes of moderate intensity exercise per week. In general, our patients exceeded the recommendation, which could be interpreted as a positive motivational effect of the HappyAir program. In addition, we observed an important relationship between mood and tiredness expressed by the patients at the end of the physical activity. This observation showed that the better the mood was, the longer the exercise period lasted; additionally, patients felt less or not tired at all when their mood was positive. This shows that the emotional and psychological states of the patients may be more important factors in achieving adherence to physical activity than the increase in physical capacity itself, corroborating the theory of the study of Mantoani [5].

### Social Support and the Role of Therapeutic Educator

Regarding the role of therapeutic educator in maintenance programs, several studies have concluded that this is a very important predictor of adherence [45,46], but not all maintenance programs include it. An important and differentiating aspect of our follow-up program was the figure of the respiratory educator/physiotherapist whom patients could contact at any time. All consultations made were classified into two categories: a need for social support or technical issues, such as with the computer web-based app, connections, or their mobile device. Motivation and support provided by the therapeutic educator were key to successful therapy and patient adherence [31].

### Behavioral Change and Its Influence on Adherence

The key that determines the difference in adopting and maintaining any habit lies in the way the initial decision making is carried out, which must be a process shared with the patient: a reasoned discussion about a process that allows that person to be aware of their current health status and ascertains their desire to maintain their health positively in the future. This happens in a dialogue and analysis about the current situation and the undesired alternative. To maintain an adequate level of physical activity in unsupervised periods, when it is very easy to become sedentary, patients must adopt behavioral changes associated with physical abilities. In that sense, the therapeutic educator has an important role to favor the commitment and maintenance of the level of physical activity adopted during the rehabilitation in the long term [34].

Both health professionals and patients consider face-to-face appointments necessary and irreplaceable by technology. It should be noted that the relationship established between the two, also known as therapeutic alliance, largely determines the success of the treatment. Scientific evidence supports the idea that the quality of the health professional–patient relationship is strongly related to patient satisfaction [12]. Thus, the HappyAir web-based app used in this study included the option of contacting the therapeutic educator, as needed, to achieve the necessary personalized support. An analysis of the records showed that approximately 65% of direct contacts requested were for assistance in resolving technical issues and were not related to clinical or social support on disease management issues.

The results were favorable in terms of adopting positive habits in self-care and from the patients’ point of view, about the support they received in clinical, social, and therapeutic guidance. Some recent studies, such as by Boer and colleagues [47], highlighted patients’ perception of the usability and support offered by mHealth systems. However, as in some of the studies reviewed [48], the therapeutic educator reported an increased workload compared with studies of periodic calls or more time-spaced follow-up.

### Quality of Life

According to published literature, during the development of maintenance programs, clinical improvements in quality of life last about 6 to 9 months [9,49]. Thus, our study conducted a follow-up of greater duration than the usual ones in the literature, where the usual follow-up period ranges from 3 to 6 months [15,50-53], and was able to verify that at 1-year follow-up the HappyAir group studied showed significant differences in health-related quality of life over time from the values presented prior to pulmonary rehabilitation until the evaluations carried out at 6 and 12 months, with a gradual improvement in their values, as shown objectively with the SGRQ and CAT questionnaires.

### Exercise Capacity, Perceived Dyspnea, and Fatigue

Patients in the HappyAir group showed a clinical improvement in exercise capacity using the 6MWT at 6-month follow-up, and they were the only group to maintain differences in this outcome from baseline to 12 months. The effect of improvement at 6 months could be attributed to our integrated care plan, since the control group did not present this outcome. But we cannot know exactly because rehabilitation itself can sometimes be responsible for this long-term effect, as shown in several studies [9,10,49]. However, the differences maintained at 12-month follow-up could be directly related to the comprehensive maintenance program, since pulmonary rehabilitation programs consisting of exercise and education have not improved the quality of life and physical capacity after 9 months of follow-up [30,54].

### Future Research

After analyzing the results of this study and comparing them with similar studies, it can be concluded that the direction to follow for the design of programs that improve adherence in the management of COPD should be focused on generating behavioral changes and better perceptions in patients. So, the idea is to act on the most essential aspect of the patients, their person and their disease, to achieve awareness of it, generate a proactive attitude, and empower the patient to make them responsible for their care.

The development and use of mHealth systems and innovative technology must be implemented and advanced, as they indicate a promising future. They should be considered as the resources needed to improve patient support and monitoring to learn more about the individual or apply a personalized approach. Moreover, they can generate better outcomes for each person in health and social support, as well as provide further information to apply innovative care and therapeutic techniques in patient management by health care teams, offering resources to those people who live in remote locations with low access to ongoing care management.

### Limitations

One of the limitations of this study is the small number of patients included. Although we calculated the sample size, the number of patients included in the control group was too small to extrapolate the results to a more heterogeneous population. However, the sample was in concordance with the majority of similar studies. Another limitation is not having assessed exercise capacity with a gold standard test, such as an incremental exercise test. We chose instead to use simple field tests commonly used in the clinical setting in order to facilitate the implementation of the program in the future. In relation to this, the long-term results achieved by patients does not reflect the increase in daily physical activity observed by the HappyAir integrated care plan. Finally, this study was designed for those patients who would adapt to using a mobile phone. If they had low-level technical skills, this would be a limitation that would need addressing with other technology or support (eg, voice technology).

### Conclusions

This study showed the development of the HappyAir integrated care plan after pulmonary rehabilitation, which uses a web-based app accessible by a mobile device and involves periodic therapeutic educator (coach) support. This has been shown to be effective in improving patient adherence to their self-care plan and treatment and consequently their state of health and attitude, with a resulting change in their perception of the disease and their engagement on their care, key factors to achieve positive health outcomes. Internet-enabled and telehealth web-based apps can serve as a means to transform and reinvent the way patients and health care professionals interact. However, this study shows the development and preliminary evaluation of a novel mHealth web-based platform in a reduced sample, which limits the generalization of our results. Further research is needed to integrate HappyAir into larger study populations with COPD.

## Appendix

Multimedia Appendix 1 HappyAir app supplementary information.

Multimedia Appendix 2 Pulmonary function, exercise capacity, and quality of life effects along the study.

Multimedia Appendix 3 Consort checklist.

## Abbreviations

6MWT: 6-Minute Walk Test
CAP FISIO: respiratory physiotherapy adherence self-report questionnaire (Spanish, Cuestionario Adherencia Percepción Physio)
CAT: Chronic Obstructive Pulmonary Disease Assessment Test
COPD: chronic obstructive pulmonary disease
GOLD: Global Initiative for Chronic Obstructive Lung Disease
mHealth: mobile health
SGRQ: St. George’s Respiratory Questionnaire
