# Supplementary material for: Development and Preliminary Evaluation of the Effects of an mHealth Web-Based Platform (HappyAir) on Adherence to a Maintenance Program After Pulmonary Rehabilitation in Patients With Chronic Obstructive Pulmonary Disease: Randomized Controlled Trial
Source: JMIR Mhealth Uhealth. 2020 Jul 31;8(7):e18465. doi: 10.2196/18465 (PMC7428903; doi:10.2196/18465)
Supplement: Multimedia Appendix 1 [file mhealth_v8i7e18465_app1.pdf]

Supplementary information.

Happyair™ APP

The Happyair™ App had a very intuitive design to facilitate its handling by all types of patients. The educator was in charge of explaining their management to the patient and their caregiver during the presential post-rehabilitation workshops. The Happyair™ application was a means of patient-educator interaction. Each patient was assigned a specialist educator in respiratory physiotherapy, who was given the opportunity to contact in case of need. The application consisted of 4 main areas, each of which in turn consisted of several items that the patient had to fill in (Figure 2).

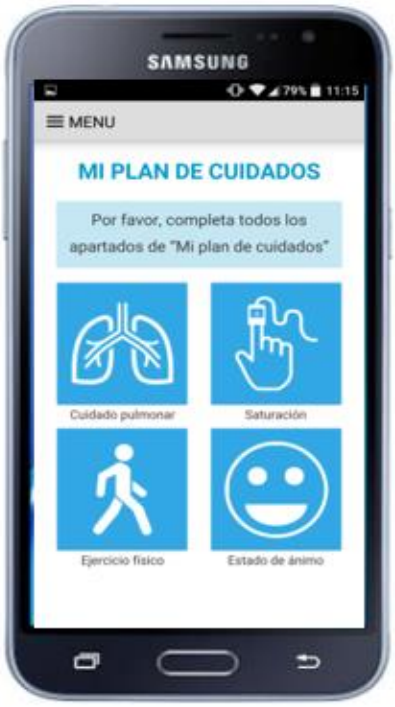

Daily self-care monitoring (PROs)	
Lung Care	Warning signs: Check list
	Respiratory Physiotherapy: Signs & Symptoms: phlegm
	Rescue Inhaler: If used, tell us why
O_2 saturation Heart Rate	Basal and post physical activity
Physical Activity	How many times did you exercise and how did you feel?
Wellness	How are you feeling today?
Educator	Direct contact to respiratory physiotherapist

Figure 2. App Happyair™.

App Happyair™ menu.

My plan: It corresponds to the weekly and monthly goals that the patient had to achieve during the follow-up period.

My Lung Care: This section showed warning signs that could lead to possible exacerbation and the patient´s adherence to pharmacological treatment and physiotherapy care: r**escue inhaler use**, **medications**, **pulmonary check** (warning signs)

My Physical Activity: The patient marked daily if he had carried out his scheduled physical activity exercises, how long he had been doing them and the sensation of breathlessness, according to the Borg scale, which he had at the end of them.

My Steps: The patient could record the number of daily steps that were recorded by the installed Google Fit application.

Sats: The patient could record his saturation in the app using a pulse oximeter supplied for this purpose.

Mood: Every day the patient could mark how he was feeling in four possible situations: cheerful, a little sad, sad and very sad.

Lovexair: Access to the Lovexair microsite, in which were located, in downloadable version, the educational contents provided to patients during the study, in addition to various information about their pathology.

The objective of the integration of this application into the daily routine of the patients, was to favour the consolidation of the acquired knowledge about their illness, as well as the maintenance of respiratory physiotherapy and physical activity exercises. The performance of all these activities should be recorded daily in the application, as well as other aspects such as taking medication. In this way, the educator was allowed to assess the warning signs of possible exacerbations or relapses, making a specific follow-up of the patient during the study.
