# Supplementary material for: Development and Preliminary Evaluation of the Effects of an mHealth Web-Based Platform (HappyAir) on Adherence to a Maintenance Program After Pulmonary Rehabilitation in Patients With Chronic Obstructive Pulmonary Disease: Randomized Controlled Trial
Source: JMIR Mhealth Uhealth. 2020 Jul 31;8(7):e18465. doi: 10.2196/18465 (PMC7428903; doi:10.2196/18465)
Supplement: Multimedia Appendix 2 [file mhealth_v8i7e18465_app2.docx]

Multimedia Appendix 2. Pulmonary function, exercise capacity, and quality of life effects along the study.

|  | CONTROL GROUP | | INTERVENTION GROUP HAPPYAIR™ | | CONTROL GROUP | | INTERVENTION GROUP HAPPYAIR™ | |
| --- | --- | --- | --- | --- | --- | --- | --- | --- |
|  | Pre-PR | Post-PR | Pre-PR | Post-PR | 6m PostRHB | 12m PostRHB | 6m PostRHB | 12m PostRHB |
| **Lung Function** |  |  |  |  |  |  |  |  |
| FEV_1_ (L) | 43.1±13.6 | - | 45±15.2 | - | - | 42.4±17 | - | 49.8±18.6 |
| FVC (L) | 72.5±24.4 | - | 78.6±22.9 | - | - | 75.9±22 | - | 73.3±21.6 |
| FEV_1_/FVC | 44.5±12.2 | - | 49±12 | - | - | 47.3±12.9 | - | 49.7±11 |
| **Quality of life** |  |  |  |  |  |  |  |  |
| CAT | 16±7.3 | 14.5±7 | 16.3±6.2 | 14.3±5.5 | 13.8±7 | 17.7±7.4 | 12.1±5.8* | 14.1±7.2 |
| SGRQ Symptom | 48.2±20.6 | 47.3±19.4 | 46.8±20.1 | 43.1±20.3 | 32.4±17.3* | 37.5±19 | 34.7±17.9* | 39.5±24.2 |
| SGRQ Activities | 69.5±18.1 | 65.7±19.1 | 67.3±21.5 | 60.3±19.5 | 66.6±21.3 | 71.4±16.3 | 60±24 | 67.6±22.4 |
| SGRQ Impact | 45.4±15.3 | 44.9±16.6 | 47.2±12.3 | 45.4±9.4 | 45.8±16.8 | 35.8±16.1* | 42.9±10.6 | 35.2±19.2* |
| SGRQ Total | 53.1±14.8 | 50.4±14.2 | 53.2±12.1 | 49.5±11.1 | 49.9±15.8 | 46.8±14 | 46.8±13.3* | 45.7±19.8* |
| EUROQOL 5D | 0.5±0.2 | 0.5±0.2 | 0.5±0.2 | 0.5±0.2 | 0.5±0.2 | 0.5±0.2 | 0.6±0.2 | 0.5±0.2 |
| VAS | 53.8±12.2 | 59.0±16.8 | 63.2±15 | 67.6±10.1 | 57.1±18.3 | 56.6±16.8 | 66.4±14.9 | 62.3±16.6 |
| **Exercise tolerance** |  |  |  |  |  |  |  |  |
| 6MWD (m) | 333.8±69.2 | 362.6±72.2 | 336.2±76.7 | 378.4±90.2* | 326.4±83.1 | 339.9±75.9 | 348.2±95.1 | 357.4±112.5 |
| 6MWT (Dysp.Post) | 3.8±2.3 | 3.4 ±1.8 | 3.8±1.7 | 2.7±1.7 | 4.6±2.5 | 4.6±2.5 | 3.4±2.1 | 3.5±2.4 |
| 6MWT (Fatig Post) | 1.8±1.7 | 1.9±1.6 | 1.1±1.0 | 1.9±1.5 | 1.7±2.3 | 1.7±2.4 | 1.1±1.0 | 1.4±2.2 |

Data presented as mean and SD. FEV1: forced expiratory volume in the first second of expiration; FVC: forced vital capacity; FEV1/FVC: quotient between forced expiratory volume in the first second of expiration and forced vital capacity; CAT: quality of life questionnaire (COPD ASSESSMENT TEST); SGRQ: respiratory questionnaire; EUROQOL 5D: quality of life questionnaire; VAS: visual analogue scale; 6MWD: distance covered in the six-minute walking test; CAP: adherence assessment questionnaire, with two dimensions, adherence and perception. * Significant differences versus pre-PR (*P<*0.05)
